# Artificial intelligence to advance acute and intensive care medicine

**DOI:** 10.1097/MCC.0000000000001150

**Published:** 2024-03-22

**Authors:** Laurens A. Biesheuvel, Dave A. Dongelmans, Paul W.G. Elbers

**Affiliations:** a Department of Intensive Care Medicine, Center for Critical Care Computational Intelligence (C4I), Amsterdam Medical Data Science (AMDS), Amsterdam Cardiovascular Science (ACS), Amsterdam Institute for Infection and Immunity (AII), Amsterdam Public Health (APH), Amsterdam UMC; b Quantitative Data Analytics Group, Department of Computer Science, Faculty of Science, Vrije Universiteit; c Department of Intensive Care Medicine, Amsterdam Public Health (APH), Amsterdam UMC, University of Amsterdam; d National Intensive Care Evaluation Foundation, Amsterdam, The Netherlands

**Keywords:** artificial intelligence, generative artificial intelligence, machine learning, predictive models

## Abstract

**Purpose of review:**

This review explores recent key advancements in artificial intelligence for acute and intensive care medicine. As artificial intelligence rapidly evolves, this review aims to elucidate its current applications, future possibilities, and the vital challenges that are associated with its integration into emergency medical dispatch, triage, medical consultation and ICUs.

**Recent findings:**

The integration of artificial intelligence in emergency medical dispatch (EMD) facilitates swift and accurate assessment. In the emergency department (ED), artificial intelligence driven triage models leverage diverse patient data for improved outcome predictions, surpassing human performance in retrospective studies. Artificial intelligence can streamline medical documentation in the ED and enhances medical imaging interpretation. The introduction of large multimodal generative models showcases the future potential to process varied biomedical data for comprehensive decision support. In the ICU, artificial intelligence applications range from early warning systems to treatment suggestions.

**Summary:**

Despite promising academic strides, widespread artificial intelligence adoption in acute and critical care is hindered by ethical, legal, technical, organizational, and validation challenges. Despite these obstacles, artificial intelligence's potential to streamline clinical workflows is evident. When these barriers are overcome, future advancements in artificial intelligence have the potential to transform the landscape of patient care for acute and intensive care medicine.

## INTRODUCTION

Acute and intensive care medicine and technological advancements have always gone hand in hand, with many innovations having indisputably contributed to substantial improvements in patient outcomes and resource allocation. However, as this decade unfolds, we find ourselves amidst an era that may truly transcend technological boundaries due to recent advancements in artificial intelligence. Given the abundant data availability, as well as the remarkable readiness to adopt new technologies, acute and intensive care medicine is exceptionally well positioned to deliver on the promise of personalized, data-driven medicine by artificial intelligence.

At its core, artificial intelligence refers to the simulation of human intelligence by computer systems. However, the term is commonly used for a wide range of technologies that involve the analysis and interpretation of data, pattern recognition and automatic decision-making. Machine learning is a prominent subset of artificial intelligence, which involves the development of algorithms that allow systems to learn from data. Typically, the development of these systems requires substantial amounts of computing power and large amounts of data. As these requirements are increasingly fulfilled, machine learning algorithms are becoming more capable of making sense of progressively complex data patterns [1].

Some of the most notable advancements artificial intelligence has brought to the medical domain are found in predictive analytics. Traditionally, acute and intensive care have mainly been reactive, with interventions typically initiated only after clinical deterioration. But predictive analytics may allow for proactive measures to mitigate potential complications or deteriorations. Examples include forecasting sepsis, respiratory distress and cardiac events, potentially contributing to improved outcomes and prognostication.

Furthermore, recent artificial intelligence innovations are paving the way for a transformation in medical imaging. For many image interpretation tasks in emergency medicine, increasingly complex *deep learning* models trained on very large amounts of digital imaging are now on par with the performance of radiologists and have shown to augment and expedite their performance [2]. Complex models can detect the subtlest anomalies that might have otherwise been missed. Thus, artificial intelligence driven image analysis tools are heading to become an indispensable tool to support radiologists to streamline prompt diagnoses and minimize errors.

However, the most profound artificial intelligence driven revolution in medicine is likely yet to come. A preview of what lies ahead in the near future is offered by the introduction of powerful models built on *transformers*, a type of neural network architecture introduced in 2017 [3]. These models can accurately analyze and extract intricacies from large bodies of unstructured text, or even images. Currently, their most widely known application is ChatGPT [4], but recent variations of transformers that were specifically trained for the medical field open a plethora of possibilities that will undoubtedly revolutionize the way we practice medicine in the coming future.

It is against this exciting background that we will explore the remarkable advancements already delivered to acute and intensive care medicine by artificial intelligence and venture into major milestones that are expected in the near future. In addition, we will also discuss vital technical, ethical and legal challenges that need to be addressed before successful implementation of artificial intelligence from emergency medical dispatch to ICUs with the ultimate goal of improving outcomes for patients and society. 

**Box 1:**
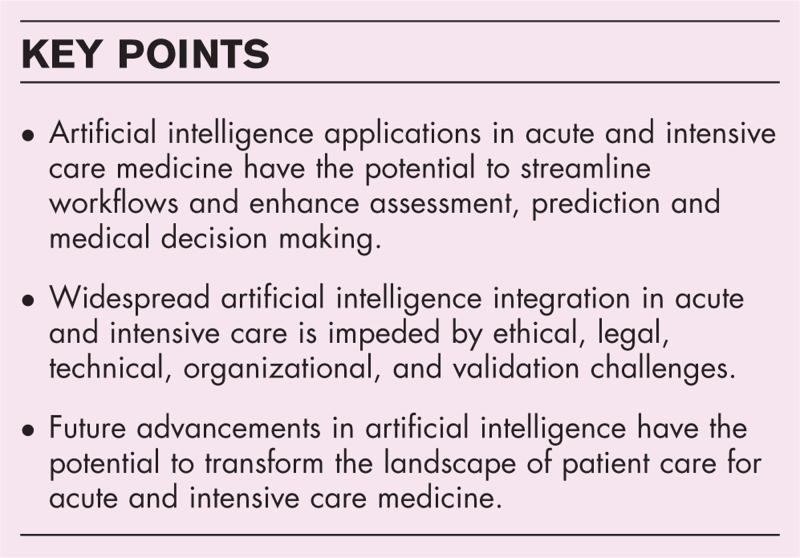
no caption available

## ARTIFICIAL INTELLIGENCE IN EMERGENCY MEDICAL DISPATCH

When a patient is involved in a medical emergency, their trajectory of medical care is initiated by a call to the emergency services. During this phone call, an emergency dispatcher is tasked with gathering important information, assessing the acuity of the situation, allocating resources and providing vocal instructions prior to handing the case over to Emergency Medical Services (EMS). Prompt and accurate evaluation is of vital importance, especially under certain circumstances when prioritization can be challenging, such as during mass casualty events or pandemics. While artificial intelligence is still in its early stages in this domain, there are some examples where it opens potential avenues to streamline assessment and decision making.

As a notable example, commercial (but still investigational) systems can serve as a second listener during the call using automatic speech recognition (ASR). Capable of extracting relevant information in real time, these systems aim to recognize signs of potentially life-threatening conditions and suggest essential follow-up questions that might otherwise be missed. Comprehensive case notes are then automatically generated, streamlining the administrative process involved.

Underlying technologies have been evaluated in a retrospective trial [5], which compared the time it takes to recognize out-of-hospital cardiac arrest (OHCA) using an artificial intelligence driven ASR system, where the artificial intelligence outperformed the dispatcher (median time to recognition 72 vs. 94 s). A similar system was assessed in a randomized clinical trial in Denmark [6], which examined the rate of dispatcher recognition of OHCA. The ASR model in use listened for signs that may indicate OHCA and alerts the dispatcher. While the machine learning model surpassed dispatcher recognition rates of OHCA (85.0 vs. 77.5%), it had a lower positive predictive value (17.8 vs. 97.4%). Among calls that the machine learning model suspected of OHCA, alerting the dispatcher did not lead to significant higher rates of recognition (93.1% in the intervention group vs. 90.5% in the control group). While these results illustrate that these technologies need further refinement to reach a level where they can reliably augment human judgment, they demonstrate notable progress using novel techniques.

## ARTIFICIAL INTELLIGENCE IN EMERGENCY DEPARTMENT TRIAGE

Efficient and adequate assessment in a timely manner is key in in the emergency department. Based on referral, patient history and vital signs, triagists assess priority. Typically, the patient is categorized on a scale ranging from nonurgent to critical, based on established protocols. While usually effective and careful, this process is dependent on human expertise and therefore not flawless.

The promise of artificial intelligence for this task lies in its potential capacity to swiftly take into account a comprehensive range of available data. This includes current history and vital signs, but also data from the medical history from the electronic medical record. The following examples highlight how taking into account this broad scope of unstructured and structured data can expedite the identification of urgent cases with more accuracy and facilitate efficient use of resources.

One avenue of artificial intelligence in emergency triage lies in outcome prediction. Chen *et al.*[7^▪^] trained a model on a retrospective dataset to predict which patients develop critical outcomes after triage, defined as in-hospital cardiac arrest (IHCA) or ICU admission. They employed structured and unstructured data and compared it to the performance of emergency physicians, showing an improved sensitivity (95 vs. 41%) and accuracy (90 vs. 67%) compared to emergency physicians, and a comparable specificity (77 vs. 78%). While the results are promising, they have not been externally validated.

A commercial but investigational model was shown to be more accurate in determining acuity on the Emergency Severity Index (ESI) than nurses in a retrospective study, using only information available at triage [8]. This study also used both structured and unstructured data. In comparison to nurses, the model was able to assign ESI acuity with an accuracy of 75.7%, as opposed to 59.8% by the nurse after retrospective labeling by expert clinicians. While the results are promising, it should be noted that, to our knowledge, no impact study has been performed yet.

In a preprint study, the same main author reported on an investigational sepsis prediction model [9] that also incorporates both structured and unstructured data to predict the probability of sepsis for a retrospective cohort. They allege that their model outperforms standard screening protocols (sensitivity of 71.09 vs. 40.8%; specificity of 94.81 vs. 95.72%). While sepsis is a widely studied outcome for many prediction models, including in the emergency department [10], this study is unique for the utilization of unstructured data.

## ARTIFICIAL INTELLIGENCE IN MEDICAL CONSULTATION IN THE EMERGENCY DEPARTMENT

Human expertise is of paramount importance during history taking and physical examination. Unfortunately, subsequent documentation of findings is a laborious process. It is estimated that physicians spend 43% of their time documenting findings [11]. Artificial intelligence can alleviate this administrative burden by serving as a second listener and automatically summarizing key findings of the medical history into the EHR. While multiple investigational commercial products offer this functionality already, no impact studies have been published to date to our knowledge.

In medical imaging, the role of artificial intelligence is already more apparent. In a 2020 survey, 30% of radiologists are already utilizing artificial intelligence models in their practice [12]. By automating assessment, the slightest anomalies can be detected with high accuracy. While a human check is still of essential importance, the advantages for accuracy and efficiency are clear [2]. When these systems become more accurate and explainable in the future, they have the capacity to significantly reduce the time between study and diagnosis.

An exciting new development in medical artificial intelligence is large multimodal generative models to potentially support the clinician in medical decision making in the future. These are very large models that take a wide range of biomedical data as input, such as unstructured clinical text and imaging, and arrive at accurate interpretable outputs in multiple domains. When these technological innovations achieve greater maturity, the use are myriad.

The first multimodal generative model was introduced in July 2023 by Google, called Med-Palm M [13^▪▪^]. Similar to the model behind ChatGPT, it is a model that has been trained on an enormous corpus of text. Med-Palm M distinguishes itself by being specially optimized using a large amount of medical data. They assessed its performance on a novel biomedical benchmark called MultiMedBench, which involved 14 medical tasks, including question answering, image interpretation and radiology report generation.

While it is a single model, it has shown to reach near state-of-the-art performance on all tasks included in this benchmark. Because it outputs interpretable language and is able to use a broad range of clinical information at once, it can be used interactively by the physician to support the process of medical decision-making. While its possibilities are clear, the authors regard Med-Palm M as a proof of concept. There remain challenges, such as technical, ethical and validation considerations, before such a model can be implemented into an EHR. But at the current pace of generative artificial intelligence advancements, it provides a promising insight of what is yet to come in the near future.

## ARTIFICIAL INTELLIGENCE IN THE ICU

For patients admitted to the ICU, a vast amount of data is routinely collected. It is therefore particularly suited for the implementation of machine learning models that support clinicians in medical decision-making, prognosis and treatment. The suitability of machine learning for the ICU is reflected by the rising amount of research being performed in this field. This surge is fueled by multiple recent efforts to make ICU research databases publicly available to researchers. Notable databases include the Medical Information Mart for Intensive Care (MIMIC) [14^▪▪^] and eICU [15] databases from the United States and AmsterdamUMCdb from The Netherlands [16]. These databases paved the way for researchers exploring the capabilities and predictive powers of machine learning.

Use cases in the ICU can be roughly categorized into early warning systems, prognosis tools, models that suggest treatment, disease phenotyping, and resource management. A 2021 systematic review [17] shows that most artificial intelligence studies in the ICU are carried out to predict complications (22.2%), followed by predicting mortality (20.6%), improving prognostic models (18.4%), and classifying sub-populations (11.7%). Other studied cases include determining physiological thresholds (4.9%), predicting the length of stay (4.4%) and alarm reduction (4.3%). Of the 494 studies included in this systematic review, only 18 were studied prospectively. In terms of technological readiness, in 2020, 93% of ICU machine learning models were at the model prototyping and development phase (phase 3-4), and only 1% reached real-time model testing (phase 6) [18].

In one of the few machine learning models for intensive care medicine that has been validated in practice, a severe sepsis prediction algorithm was assessed in a single-center randomized clinical trial. The average hospital length of stay (13.0 vs. 10.3 days; *P* = 0.042) and in-hospital mortality (8.96 vs. 21.3%; *P* = 0.018) were significantly reduced for the experimental group [19]. However, it is generally expected that more models will reach the bedside of critically ill patients in the near future. A promising example is an investigational model predicting which patients can be discharged safely from intensive care [20], which was recently shown to retain performance following external validation and retraining [21^▪^].

Particularly interesting are studies that incorporate a technique called reinforcement learning in the ICU, which is a technique within machine learning that is concerned with automatically taking or suggesting learned optimal actions within a dynamic environment to maximize a certain outcome [22]. More specifically, these are algorithms that are capable of providing sequential suggestions for treatment within the ICU, such as ventilator settings or the optimal balance between vasopressors and fluids. While the results of reinforcement learning algorithms in retrospective databases are promising, there are no studies published that study performance in a prospective setting [23^▪^].

## CHALLENGES

The paucity of model integration into acute critical care relative to the very large number of produced papers is striking. This is partly explained by the relative ease of training a model on retrospective data vs. the laborious steps involved in implementation [17]. Major obstacles include ethical, legal, technical, organizational, and validation challenges.

Ethical challenges include balancing patient privacy and progress as well as ensuring that models generalize well to minority groups or patients from healthcare systems that are differently structured. Legal challenges include well intended but rather strict regulations hampering model implementation in clinical practice. Implementation requires trust, which is optimally achieved by training and validating models that are well tolerated, transparent, and explainable. Comprehensibility of models is favorable, but often limits development to relatively simple ‘explainable’ models as opposed to complex ‘black box’ variants. Transparency is often hampered by the proprietary nature of implemented models. Additionally, those models that have made it to the bedside often lack external validation and impact studies and may not be as accurate in real-world practice [24].

Other reasons for the gap between theoretical research and practical clinical application are explained by lack of actionability. Predicting any outcome is not useful if it does not alter clinical decision making. In addition, a model that gives too many predictions or warnings that don’t require immediate attention can lead to ‘alarm fatigue’ [25], which might cause important events to be missed. Favorably, such warnings are prioritized based on urgency. Solving these challenges requires initiatives that promote collaboration between data scientists and healthcare professionals, such as interdisciplinary forums and joint development teams. Close collaboration from the outset of project design encourages that models are developed with a clear comprehension of clinical value, practicality, and technological feasibility.

Finally, when underlying technologies of artificial intelligence further mature, the future of advanced care likely involves close collaboration between artificial intelligence and the healthcare professional to augment the medical decision-making process. To prepare for this era, and to promote making informed decisions based on artificial intelligence, healthcare training programs should be adapted to cover interpretation of artificial intelligence generated data, and cover awareness about potential biases and limitations.

## CONCLUSION

Over recent years, rising interest in artificial intelligence has clearly sparked academic initiatives to study its capabilities within acute and critical care. However, the gap between research and bedside implementation remains large, highlighting the major challenges involved in this process. While models that streamline clinical documentation are already here to reduce the administrative burden for healthcare providers, a true overhaul by artificial intelligence may still be some years away. Nevertheless, artificial intelligence continues to advance at lightning speed and might guide us toward a new era in acute and critical medicine sooner than expected.

## Acknowledgements

*None.*

### Financial support and sponsorship

*Amsterdam UMC funding only.*

### Conflicts of interest

*Amsterdam UMC receives royalties from the artificial intelligence scale-up company Pacmed.*

## REFERENCES AND RECOMMENDED READING

Papers of particular interest, published within the annual period of review, have been highlighted as:

▪ of special interest
▪▪ of outstanding interest
